# Supplementary material for: Improving the mental health of women intimate partner violence survivors: Findings from a realist review of psychosocial interventions
Source: PLoS One. 2022 Mar 17;17(3):e0264845. doi: 10.1371/journal.pone.0264845 (PMC8929660; doi:10.1371/journal.pone.0264845)
Supplement: S1 File — (DOCX) [file pone.0264845.s001.docx]

**S1 Scoping Articles**

**Review Articles used in Exploratory Scoping**

1. Arroyo, K., Lundahl, B., Butters, R., Vanderloo, M., & Wood, D. S. (2017). Short-Term Interventions for Survivors of Intimate Partner Violence: A Systematic Review and Meta-Analysis. Trauma, Violence, and Abuse, 18(2), 155–171. https://doi.org/10.1177/1524838015602736
2. Bourey, C., Williams, W., Bernstein, E. E., & Stephenson, R. (2015). Systematic review of structural interventions for intimate partner violence in low- and middle-income countries: Organizing evidence for prevention Health behavior, health promotion and society. BMC Public Health, 15(1). https://doi.org/10.1186/s12889-015-2460-4
3. Gilbert, L., Raj, A., Hien, D., Stockman, J., Terlikbayeva, A., & Wyatt, G. (2015). Targeting the SAVA (Substance Abuse, Violence, and AIDS) Syndemic Among Women and Girls: A Global Review of Epidemiology and Integrated Interventions. Journal of Acquired Immune Deficiency Syndromes, 69, S118–S127. https://doi.org/10.1097/QAI.0000000000000626
4. Hackett, S., McWhirter, P. T., & Lesher, S. (2015). The Therapeutic Efficacy of Domestic Violence Victim Interventions. Trauma, Violence, and Abuse, 17(2), 123–132. https://doi.org/10.1177/1524838014566720
5. Jahanfar, S., Howard, L. M., & Medley, N. (2014, November 12). Interventions for preventing or reducing domestic violence against pregnant women. Cochrane Database of Systematic Reviews. John Wiley and Sons Ltd. https://doi.org/10.1002/14651858.CD009414.pub3
6. Kirk, L., Terry, S., Lokuge, K., & Watterson, J. L. (2017). Effectiveness of secondary and tertiary prevention for violence against women in low and low-middle income countries: A systematic review. BMC Public Health, 17(1). https://doi.org/10.1186/s12889-017-4502-6
7. Rivas, C., Ramsay, J., Sadowski, L., Davidson, L. L., Dunne, D., Eldridge, S., … Feder, G. (2015, December 3). Advocacy interventions to reduce or eliminate violence and promote the physical and psychosocial well-being of women who experience intimate partner abuse. Cochrane Database of Systematic Reviews. John Wiley and Sons Ltd. https://doi.org/10.1002/14651858.CD005043.pub3
8. Sprague, S., Scott, T., Garibaldi, A., Bzovsky, S., Slobogean, G. P., McKay, P., … Swaminathan, A. (2017). A scoping review of intimate partner violence assistance programmes within health care settings. European Journal of Psychotraumatology. Taylor and Francis Ltd. https://doi.org/10.1080/20008198.2017.1314159
9. Warshaw, C., Sullivan, C. M., & Rivera, E. a. (2013). A systematic review of trauma-focused interventions for domestic violence survivors. National Center on Domestic Violence, Trauma & Mental Health, (February), 1–27. Retrieved from http://www.nationalcenterdvtraumamh.org/wp-content/uploads/2013/03/NCDVTMH_EBPLitReview2013.pdf
10. Anderson, J. C., Campbell, J. C., & Farley, J. E. (2013). Interventions to address HIV and intimate partner violence in sub-saharan Africa: A review of the literature. Journal of the Association of Nurses in AIDS Care, 24(4), 383–390. https://doi.org/10.1016/j.jana.2013.03.003
