# Supplementary material for: Improving the mental health of women intimate partner violence survivors: Findings from a realist review of psychosocial interventions
Source: PLoS One. 2022 Mar 17;17(3):e0264845. doi: 10.1371/journal.pone.0264845 (PMC8929660; doi:10.1371/journal.pone.0264845)
Supplement: S2 File — (DOCX) [file pone.0264845.s002.docx]

**S2 Preliminary Conceptual Model**

**Conceptual Model Developed after Scoping and Prior to Full Review.**

Therapeutic Rapport

Referral/ Access to services

Empathy

Affirmation

Active Listening

Validation

Safety

Discuss Treatment Options

Case Management

Relational

Ability to Assess Risks

Problem Solving Skills

Ability to set and Assess Goals

Crisis Management

Negotiation Skills

Financial Capacity Building

Other Behavioural Skills

Develop New Patterns and Skills

Behavioural

Cognitive

Change/ Shift in Thought Processes / Patterns

Increased Knowledge / Awareness

Knowledge and Understanding

Psycho-education

Insight Building

Coping Strategy Enhancement

Trauma Processing

Motivational Enhancement

Cognitive Restructuring

Revaluating Subjectivity (Self-Self)

Emotional Regulation

Self-Compassion

Anxiety Reduction

Increased Hope

Reduced Shame / Stigma

Mindfulness (behaviours / attitudes)

Engaging with Values, Beliefs and Ideologies

Revaluating Self-Society Relations

Emotional / Spiritual

Social Support

Social Engagement

Family relationship Strengthening

Family capacity building

Community capacity Building

Changes in Policies/ regulations

Intervention

Social

Increased Support

Interpersonal / Social Change
