# Supplementary material for: Improving the mental health of women intimate partner violence survivors: Findings from a realist review of psychosocial interventions
Source: PLoS One. 2022 Mar 17;17(3):e0264845. doi: 10.1371/journal.pone.0264845 (PMC8929660; doi:10.1371/journal.pone.0264845)
Supplement: S3 File — (DOCX) [file pone.0264845.s003.docx]

**S3 Final Reviews Included**

**Final Reviews Included in the Realist Synthesis**

1. Cynthia R. Young, Diane M. Arnos & Lynn T. Matthews (2019) A scoping review of interventions to address intimate partner violence in sub-Saharan African healthcare, Global Public Health, 14:9, 1335-1346, DOI: 10.1080/17441692.2019.1567802
2. Tol, W.A., Murray, S.M., Lund, C. et al. (2019) Can mental health treatments help prevent or reduce intimate partner violence in low- and middle-income countries? A systematic review. BMC Women's Health 19, 34 . https://doi.org/10.1186/s12905-019-0728-z
3. Schwab-Reese, L. M., & Renner, L. M. (2018). Screening, management, and treatment of intimate partner violence among women in low-resource settings. Women’s Health (London, England).<https://doi.org/10.1177/1745506518766709>
4. Warshaw, C., Sullivan, C. M., & Rivera, E. a. (2013). A systematic review of trauma-focused interventions for domestic violence survivors. National Center on Domestic Violence, Trauma & Mental Health, (February), 1–27. Retrieved from<http://www.nationalcenterdvtraumamh.org/wp-content/uploads/2013/03/NCDVTMH_EBPLitReview2013.pdf>
5. Arroyo, K., Lundahl, B., Butters, R., Vanderloo, M., & Wood, D. S. (2017). Short-Term Interventions for Survivors of Intimate Partner Violence: A Systematic Review and Meta-Analysis. Trauma, Violence, and Abuse, 18(2), 155–171.<https://doi.org/10.1177/1524838015602736>
6. Rivas, C., Ramsay, J., Sadowski, L., Davidson, L. L., Dunne, D., Eldridge, S., Feder, G. (2015). Advocacy interventions to reduce or eliminate violence and promote the physical and psychosocial well-being of women who experience intimate partner abuse. Cochrane Database of Systematic Reviews. John Wiley and Sons Ltd.<https://doi.org/10.1002/14651858.CD005043.pub3>
7. Trabold, N., McMahon, J., Alsobrooks, S., Whitney, S., & Mittal, M. (2018). A Systematic Review of Intimate Partner Violence Interventions: State of the Field and Implications for Practitioners. Trauma, Violence, and Abuse.<https://doi.org/10.1177/1524838018767934>
8. Rivas C., Vigurs C., Cameron, J., Yeo, L. (2019). A realist review of which advocacy interventions work to help abused women, and under what circumstances, and which women are likely to benefit. Cochrane Database of Systematic Reviews. John Wiley & Sons, Ltd.<https://doi.org/10.1002/14651858.CD013135.pub2>
9. Hackett, S., McWhirter, P. T., & Lesher, S. (2015). The Therapeutic Efficacy of Domestic Violence Victim Interventions. Trauma, Violence, and Abuse, 17(2), 123–132.<https://doi.org/10.1177/1524838014566720>
10. Shea, B., Nahwegahbow, A., & Andersson, N. (2010). Reduction of Family Violence in Aboriginal Communities: A Systematic Review of Interventions and Approaches. Pimatisiwin, 8(2), 35–60.
11. Kirk, L., Terry, S., Lokuge, K., & Watterson, J. L. (2017). Effectiveness of secondary and tertiary prevention for violence against women in low and low-middle income countries: a systematic review. BMC public health, 17(1), 622.<https://doi.org/10.1186/s12889-017-4502-6>
12. Karakurt, G., Whiting, K., van Esch, C., Bolen, S. D., & Calabrese, J. R. (2016). Couples Therapy for Intimate Partner Violence: A Systematic Review and Meta-Analysis. Journal of Marital and Family Therapy, 42(4), 567–583.<https://doi.org/10.1111/jmft.12178>
13. Semahegn, A., Torpey, K., Manu, A., Assefa, N., Tesfaye, G., & Ankomah, A. (2019, July 1). Are interventions focused on gender-norms effective in preventing domestic violence against women in low and lower-middle income countries? A systematic review and meta-analysis. Reproductive Health. BioMed Central Ltd.<https://doi.org/10.1186/s12978-019-0726-5>
14. Anderson, K., & van Ee, E. (2018, September 7). Mothers and children exposed to intimate partner violence: a review of treatment interventions. International Journal of Environmental Research and Public Health. MDPI AG.<https://doi.org/10.3390/ijerph15091955>
15. Stylianou, A. M. (2018). Economic Abuse Within Intimate Partner Violence: A Review of the Literature. Violence and Victims, 33(1), 3–22.<https://doi.org/10.1891/0886-6708.33.1.3>
16. Barner, J. R., & Carney, M. M. (2011). Interventions for Intimate Partner Violence: A Historical Review. Journal of Family Violence, 26(3), 235–244.<https://doi.org/10.1007/s10896-011-9359-3>
17. Nolan, C. R. (2016). Bending without breaking: A narrative review of trauma-sensitive yoga for women with PTSD. Complementary Therapies in Clinical Practice, 24, 32–40.<https://doi.org/10.1016/j.ctcp.2016.05.006>
18. Rees, K., Zweigenthal, V., & Joyner, K. (2014). Health sector responses to intimate partner violence: A literature review. African Journal of Primary Health Care and Family Medicine, 6(1).<https://doi.org/10.4102/phcfm.v6i1.712>
19. Duffy, L., Adams, J., Sibbritt, D., & Loxton, D. (2014). Complementary and alternative medicine for victims of intimate partner abuse: A systematic review of use and efficacy. Evidence-Based Complementary and Alternative Medicine. Oxford University Press.<https://doi.org/10.1155/2014/963967>
20. Labarre, M., Brodeur, N., Roy, V., & Bousquet, M. A. (2019, December 1). Practitioners’ Views on IPV and Its Solutions: An Integrative Literature Review. Trauma, Violence, and Abuse. SAGE Publications Ltd.<https://doi.org/10.1177/1524838017728709>
21. Leite, T. H., De Moraes, C. L., Marques, E. S., Caetano, R., Braga, J. U., & Reichenheim, M. E. (2019). Women economic empowerment via cash transfer and microcredit programs is enough to decrease intimate partner violence? Evidence from a systematic review. Cadernos de Saude Publica. Fundacao Oswaldo Cruz.<https://doi.org/10.1590/0102-311X00174818>
22. Pathak, N., Dhairyawan, R., & Tariq, S. (2019). The experience of intimate partner violence among older women: A narrative review. Maturitas, 121, 63–75.<https://doi.org/10.1016/j.maturitas.2018.12.011>
23. Avanti Adhia, Bizu Gelaye, Lauren E. Friedman, L. Y. Marlow, James A. Mercy & Michelle A. Williams (2019) Workplace interventions for intimate partner violence: A systematic review, Journal of Workplace Behavioral Health, 34:3, 149-166, DOI: 10.1080/15555240.2019.1609361
24. Sprague, S., Scott, T., Garibaldi, A., Bzovsky, S., Slobogean, G. P., McKay, P., … Swaminathan, A. (2017). A scoping review of intimate partner violence assistance programmes within health care settings. European Journal of Psychotraumatology. Taylor and Francis Ltd.<https://doi.org/10.1080/20008198.2017.1314159>
25. Anderson, E. J., Krause, K. C., Meyer Krause, C., Welter, A., McClelland, D. J., Garcia, D. O., … Koss, M. P. (2019). Web-Based and mHealth Interventions for Intimate Partner Violence Victimization Prevention: A Systematic Review. Trauma, Violence, and Abuse.<https://doi.org/10.1177/1524838019888889>
26. Kirst, M., Zhang, Y. J., Young, A., Marshall, A., O’Campo, P., & Ahmad, F. (2012, October). Referral to Health and Social Services for Intimate Partner Violence in Health Care Settings: A Realist Scoping Review. Trauma, Violence, and Abuse.<https://doi.org/10.1177/1524838012454942>
27. Ogunsiji, O., & Clisdell, E. (2017, May 4). Intimate partner violence prevention and reduction: A review of literature. Health Care for Women International. Taylor and Francis Inc.<https://doi.org/10.1080/07399332.2017.1289212>
28. Tirado-Muñoz, J., Gilchrist, G., Farré, M., Hegarty, K., & Torrens, M. (2014). The efficacy of cognitive behavioural therapy and advocacy interventions for women who have experienced intimate partner violence: A systematic review and meta-analysis. Annals of Medicine, 46(8), 567–586.<https://doi.org/10.3109/07853890.2014.941918>
29. Keynejad, R. C., Hanlon, C., & Howard, L. M. (2020). Psychological interventions for common mental disorders in women experiencing intimate partner violence in low-income and middle-income countries: a systematic review and meta-analysis. The Lancet Psychiatry, 7(2), 173–190.<https://doi.org/10.1016/S2215-0366(19)30510-3>
30. Shorey, R. C., Tirone, V., & Stuart, G. L. (2014). Coordinated community response components for victims of intimate partner violence: A review of the literature. Aggression and Violent Behavior. Elsevier Ltd.<https://doi.org/10.1016/j.avb.2014.06.001>
31. Tappis H, Freeman J, Glass N, Doocy S. Effectiveness of Interventions, Programs and Strategies for Gender-based Violence Prevention in Refugee Populations: An Integrative Review. PLOS Currents Disasters. 2016 Apr 19 . Edition 1. doi: 10.1371/currents.dis.3a465b66f9327676d61eb8120eaa5499.
32. Alvarez, C. P., Davidson, P. M., Fleming, C., & Glass, N. E. (2016, August 1). Elements of effective interventions for addressing intimate partner violence in Latina women: A systematic review. PLoS ONE. Public Library of Science.<https://doi.org/10.1371/journal.pone.0160518>
33. Hampton, R. L., Lataillade, J. J., Dacey, A., & Marghi, J. R. (2008). Evaluating domestic violence interventions for black women. Journal of Aggression, Maltreatment and Trauma.<https://doi.org/10.1080/10926770801925759>
34. Hardesty, J. L., & Ogolsky, B. G. (2020). A Socioecological Perspective on Intimate Partner Violence Research: A Decade in Review. Journal of Marriage and Family, 82(1), 454–477.<https://doi.org/10.1111/jomf.12652>
35. Schmidt, I. D. (2014). Addressing ptsd in low-income victims of intimate partner violence: Moving toward a comprehensive intervention. Social Work (United States), 59(3), 253–260.<https://doi.org/10.1093/sw/swu016>
36. Lourenço, R. G., Fornari, L. F., Santos, D. L. A. D., & Fonseca, R. M. G. S. da. (2019, January 1). Community interventions related to intimate partner violence among adolescents: scope review. Revista Brasileira de Enfermagem. NLM (Medline).<https://doi.org/10.1590/0034-7167-2018-0586>
37. Bourey, C., Williams, W., Bernstein, E. E., & Stephenson, R. (2015). Systematic review of structural interventions for intimate partner violence in low- and middle-income countries: Organizing evidence for prevention Health behavior, health promotion and society. BMC Public Health, 15(1). https://doi.org/10.1186/s12889-015-2460-4
38. Gilbert, L., Raj, A., Hien, D., Stockman, J., Terlikbayeva, A., & Wyatt, G. (2015). Targeting the SAVA (Substance Abuse, Violence, and AIDS) Syndemic Among Women and Girls: A Global Review of Epidemiology and Integrated Interventions. Journal of Acquired Immune Deficiency Syndromes, 69, S118–S127.<https://doi.org/10.1097/QAI.0000000000000626>
39. Jahanfar, S., Howard, L. M., & Medley, N. (2014, November 12). Interventions for preventing or reducing domestic violence against pregnant women. Cochrane Database of Systematic Reviews. John Wiley and Sons Ltd.<https://doi.org/10.1002/14651858.CD009414.pub3>
40. Anderson, J. C., Campbell, J. C., & Farley, J. E. (2013). Interventions to address HIV and intimate partner violence in sub-saharan Africa: A review of the literature. Journal of the Association of Nurses in AIDS Care, 24(4), 383–390.<https://doi.org/10.1016/j.jana.2013.03.003>
41. Hahn, S. A., & Postmus, J. L. (2014). Economic Empowerment of Impoverished IPV Survivors: A Review of Best Practice Literature and Implications for Policy. Trauma, Violence, and Abuse, 15(2), 79–93.<https://doi.org/10.1177/1524838013511541>
42. Marshall, K. J., Fowler, D. N., Walters, M. L., & Doreson, A. B. (2018, October 1). Interventions that Address Intimate Partner Violence and HIV Among Women: A Systematic Review. AIDS and Behavior. Springer New York LLC.<https://doi.org/10.1007/s10461-017-2020-2>
43. Choo, E. K., Gottlieb, A. S., DeLuca, M., Tape, C., Colwell, L., & Zlotnick, C. (2015). Systematic review of ED-based intimate partner violence intervention research. Western Journal of Emergency Medicine. eScholarship.<https://doi.org/10.5811/westjem.2015.10.27586>
44. Van Parys, A. S., Verhamme, A., Temmerman, M., & Verstraelen, H. (2014, January 17). Intimate partner violence and pregnancy: A systematic review of interventions. PLoS ONE. Public Library of Science.<https://doi.org/10.1371/journal.pone.0085084>
45. Colombini, M., Mayhew, S., & Watts, C. (2008, August). Health-sector responses to intimate partner violence in low- and middle-income settings: A review of current models, challenges and opportunities. Bulletin of the World Health Organization.<https://doi.org/10.2471/BLT.07.045906>
46. Sabri B, Gielen A. Integrated Multicomponent Interventions for Safety and Health Risks Among Black Female Survivors of Violence: A Systematic Review [published online ahead of print, 2017 Jan 1]. Trauma Violence Abuse. 2017;1524838017730647. doi:10.1177/1524838017730647
47. Ramon, S., Vakalopoulou, A., Lloyd, M., Rolle, L., Roszcynskya-Michta, J., & Videmsek, P. (2015). Understanding the connections between intimate partner domestic violence and mental health within the European context: Implications for innovative practice. Dialogue in Praxis. 4(17), 01-21.<https://iris.unito.it/retrieve/handle/2318/1650398/365366/FILE%20ROLLE%20PRAXIS.pdf>
48. Narain, T. & Adcock, L. (2017) Peer Support Programs for Adults Who Have Experienced Sexual Assault, Abuse, Harassment, or Misconduct: A Review of Clinical Effectiveness and Guidelines. Canadian Agency for Drugs and Technologies in Health. Ottawa, ON.
49. Bair-Merritt, M. H., Lewis-O’Connor, A., Goel, S., Amato, P., Ismailji, T., Jelley, M., … Cronholm, P. (2014). Primary care-based interventions for intimate partner violence: A systematic review. American Journal of Preventive Medicine, 46(2), 188–194.<https://doi.org/10.1016/j.amepre.2013.10.001>
50. Hegarty, K., & Tarzia, L. (2019, January 1). Identification and Management of Domestic and Sexual Violence in Primary Care in the #MeToo Era: an Update. Current Psychiatry Reports. Current Medicine Group LLC 1.<https://doi.org/10.1007/s11920-019-0991-6>
51. Taft, C. T., Bryant-Davis, T., Woodward, H. E., Tillman, S., & Torres, S. E. (2009, January). Intimate partner violence against African American women: An examination of the socio-cultural context. Aggression and Violent Behavior. https://doi.org/10.1016/j.avb.2008.10.001
52. Gierisch, J. M., Shapiro, A., Grant, N., King, H. A., McDuffie, J. R., & Williams, J. W. Jr. (2013). Intimate partner violence: Prevalence among U.S. military veterans and active duty servicemembers and a review of intervention approaches. Department of Veterans Affairs Health Services Research and Development Service (pp. 1–83). Retrieved from<https://www.hsrd.research.va.gov/publications/esp/partner_violence.p>
53. Tankard, M. E., & Iyengar, R. (2018). Economic Policies and Intimate Partner Violence Prevention: Emerging Complexities in the Literature. Journal of Interpersonal Violence, 33(21), 3367–3387.<https://doi.org/10.1177/0886260518798354>
54. Tol, W. A., Stavrou, V., Greene, M. C., Mergenthaler, C., Van Ommeren, M., & García Moreno, C. (2013). Sexual and gender-based violence in areas of armed conflict: A systematic review of mental health and psychosocial support interventions. Conflict and Health, 7(1).<https://doi.org/10.1186/1752-1505-7-16>
55. Hegarty, K., Tarzia, L., Hooker, L., & Taft, A. (2016, September 2). Interventions to support recovery after domestic and sexual violence in primary care. International Review of Psychiatry. Taylor and Francis Ltd.<https://doi.org/10.1080/09540261.2016.1210103>
56. Ebony Rempel, Lorie Donelle, Jodi Hall & Susan Rodger (2019) Intimate partner violence: a review of online interventions, Informatics for Health and Social Care, 44:2, 204-219, DOI: 10.1080/17538157.2018.1433675
57. Armenti, N. A., & Babcock, J. C. (2016). Conjoint treatment for intimate partner violence: A systematic review and implications. Couple and Family Psychology: Research and Practice, 5(2), 109–123.<https://doi.org/10.1037/cfp0000060>
58. McDougal, L., Klugman, J., Dehingia, N., Trivedi, A., & Raj, A. (2019). Financial inclusion and intimate partner violence: What does the evidence suggest? PLoS ONE, 14(10).<https://doi.org/10.1371/journal.pone.0223721>
59. Caponnetto, P., Maglia, M., Pistritto, L., Ferlito, S., & Cannella, M. C. (2019). Family violence and its psychological management at the Emergency Department: A review. Health psychology research, 7(2), 8558. https://doi.org/10.4081/hpr.2019.8558
60. Burke, J. G., O’Malley, T. L., Folb, B., Hagen, C. A., & Rabinovich, B. A. (2019). Conceptual Factors That Support Safety Behaviors Among Abuse Survivors: A Literature Review. Partner Abuse, 10(4), 452–482.<https://doi.org/10.1891/1946-6560.10.4.452>
