# Supplementary material for: Improving the mental health of women intimate partner violence survivors: Findings from a realist review of psychosocial interventions
Source: PLoS One. 2022 Mar 17;17(3):e0264845. doi: 10.1371/journal.pone.0264845 (PMC8929660; doi:10.1371/journal.pone.0264845)
Supplement: S4 File — (DOCX) [file pone.0264845.s004.docx]

**S4 CMO Tables**

**CMO Tables for Mechanisms Detailed in the Review**

| **Resource, Reasoning, Outcomes table** | |  |
| --- | --- | --- |
| ***Design and Delivery*** | | |
| **Appropriate setting** | | |
| **Resources** | **Reasoning** | **Outcomes** |
| -Accessible locations relevant to women’s lives  -Private and secure spaces  -Support for families in perinatal period with the transition to parenthood  -Short waiting times for appointments  -Safe and anonymous systems for reporting violence and seeking help | -Safe and familiar settings decrease anxiety around disclosure  -Prevention of partner suspicion  -Increased survivor sense of safety and security | -Increased reach of interventions to low-income and marginalised groups  - Intervention acceptance, uptake and engagement by survivors  - Improvements in reproductive and mental health, including decreased anxiety  - Reduction in violence |
| **Resources** | **Reasoning** | **Outcomes** |
| - Interventions adapted and integrated into existing care or work models  - Institutional support for IPV interventions | - Perceived to be less burdensome and more feasible by providers  - Increased confidence of providers in delivering interventions | - Improved survivor attitudes towards interventions  - Improved disclosure from survivors |
| **IPV tailored / trauma informed** | | |
| **Resources** | **Reasoning** | **Outcomes** |
| - Interventions more sensitive to survivor needs  - Strategies to manage trauma symptoms | - Increased self-esteem  - Increased sense of control over the situation | Intermediary  - Increased ability and willingness to participate in interventions  Final  - Reduced distress  - Improved symptoms of PTSD, depression, anxiety |
| In LMICs where resources are unavailable to tailor existing psychological interventions for common mental disorders, survivors might benefit as much as women not experiencing intimate partner violence from these more generic psychological interventions, even when delivered by non-clinically trained facilitators, when they include the following specific elements: identifying social support; problem-solving; identifying or eliciting affect (or both); linking affect to events; and identifying thoughts, and the following non-specific elements: empathy; collaboration; active listening; normalising treatment or symptoms (or both); and involving significant others. | | |
| **Individually adapted** | | |
| **Resources** | **Reasoning** | **Outcomes** |
| - Delivered through individual sessions  - Interventions meet unique and evolving needs of survivors  - Sessions planned to fit with survivor priorities, responsibilities and goals  - Survivors’ safety to engage carefully considered at each stage | - Unique needs met  - Individual strengths for recovery drawn-upon  - Increased self-esteem | Intermediary  - More meaningful changes in outcomes  - Improved emotional wellbeing  -Improved safety  Final  - Increased life functioning  - Decreased depression and distress  - Reduced PTSD  - Reduced IPV recurrence |
| **Culturally sensitive** | | |
| **Resources** | **Reasoning** | **Outcomes** |
| - Consider and directly address cultural influences and make sense of and addresses women’s stressors from a cultural perspective  - Assist women to access resources that may not be available to them due to their cultural or social group  - Adapted to include participants’ primary language, idioms and cultural concepts  - Engaging communities in building culturally-appropriate responses to IPV  - Recovery grounded in community values and traditional healing practices | - Increased participant trust in interventions  - Behaviour change  - Individual and community empowerment to address underlying causes of violence  - Greater resonance and relevance of interventions for community members  - Increased motivation and empowerment of survivors  - Interventions and support that resonate with survivor goals | Intermediary  - Reduced barriers to entry  - Improved uptake  - Increased engagement  Final  - Protection against further harm through structural violence  - Improved community accountability mechanisms |
| The prevalence of harmful stereotypes about survivors from particular racial or socio-economic groups can undermine the ability of women to participate in interventions and treatments. Interventions that are not contextually and culturally adapted to include an understanding of these stereotypes and specific related barriers will have poor uptake, retention, and outcomes. | | |
| **Improved delivery and training** | | |
| **Resources** | **Reasoning** | **Outcomes** |
| - Tools and approaches for identifying and managing IPV  - Streamlined process of training  - Written procedures and protocols  - Content introduced in line with feasibility of training and support | - Increased provider self-efficacy in responding to IPV  - Increased provider receptivity to introduction of mental health components in interventions | - Increased provider ability to provide effective responses to IPV disclosure  - Increased disclosure from survivors |
| **Resources** | **Reasoning** | **Outcomes** |
| - Broad definitions of abuse  - Confidential, respectful and non-judgemental approaches to discussing IPV  - Active listening and empathy  - Direct, open-ended, behaviourally anchored and non-stigmatising questions about abuse  - Culture of gender equality promoted  - Multiple opportunities for disclosure over time | - Promotion of empowerment and peer support among providers  - Trust and rapport built with providers | - Increased disclosure from survivors |
| **Multilevel & multi-layered interventions** | | |
| **Resources** | **Reasoning** | **Outcomes** |
| - Services that meet specific, interrelated needs  - Integrated components addressing multiple interrelated needs, particularly substance abuse, HIV and IPV with trauma-informed approaches | - Underlying difficulties faced as a result of multiple traumas recognised  - Pre-existing relationships of trust | Intermediary  - Increased acceptability of interventions to survivors  - Increased effectiveness compared to single-focus interventions  Final  - Multiple gains achieved (improved medication adherence, reduced risky sexual behaviours, reduced HIV transmission, improvements in substance use disorders, symptoms of PTSD) |
| **Increased community and family engagement** | | |
| **Resources** | **Reasoning** | **Outcomes** |
| - Engaging communities to build on local capacity and increase awareness of interventions  - Community partnerships and participatory approaches in development and implementation of interventions  - Community-wide and community generated solutions | - Improved individual and community belief in the possibility of assistance and change  - Improved community acceptance, understanding and ownership of interventions  - Reduced survivor fear of loss of community support networks when engaging with external agencies  - Communities empowered to hold perpetrators responsible, support survivors and ensure programmes can run | Intermediary  - Increased likelihood of survivors being able to seek help  - Increased individual empowerment and community strength  - Increased survivor safety  Final  - Reduced risk of IPV, family violence, sexual assault and stalking  - Reduced survivor distress  - Fosters healing |
| **Resources** | **Reasoning** | **Outcomes** |
| - Campaigns aimed at changing gender norms associated with gender-based violence  - Education and awareness raising on family concerns for own safety, reducing blaming survivor and knowledge on how to help  - Creating a supportive family environment | - Shifts in traditional attitudes about gender inequality and IPV acceptability  - Increased ability to cope  - Encouragement for survivors from family members, including children | Intermediary  - Increased outreach of interventions to vulnerable populations  - Improved community receptivity to interventions  - Increased help-seeking and participation in interventions  - Increased social support for survivors  Final  - Reductions in gender-based violence |
| **Intervention dose** | | |
| **Resources** |  | **Outcomes** |
| - Brief interventions that still consist of multiple sessions  - Ability to address complex nature of IPV and multiple needs of survivors |  | - Reduced distress  - Increased self-esteem  - Improved PTSD, depression  - Improved life functioning  - Greater improvements in mental health and reductions in violence (compared to shorter interventions)  - Reduced risk of physical IPV  - Reduced PTSD and depression symptoms |
| The strength of impact from interventions is often seen to attenuate over time in follow-ups with participants. This may either be due to survivors entering interventions at a “high crisis” stage and regressing to the mean with the passage of time, or it may be that gains are lost because survivors return to more problematic patterns of thought and behaviours without ongoing treatment, booster sessions or specific provisions in treatment that promote long term retention of gains. | | |
| ***Intervention Components*** | | |
| **Access to physical resources / services** | | |
| **Improved disclosure and referral** | | |
| **Resources** | **Reasoning** | **Outcomes** |
| - Supportive, non-judgmental, confidential, respectful and trusting relationships between provider and survivor  - Provider understanding and respect around survivor decisions  - Training for service providers, institutional policies on IPV referrals  - Access to convenient and immediate onsite referrals, onsite IPV-advocates  - referrals from healthcare providers to community-based organisations  - Ongoing support through referrals across a range of services | - Increased comfort disclosing violence  - Increased trust  - Increased safety behaviours  - Service providers motivated to make referrals | Intermediary  - Increased acceptability of interventions and uptake of services  - Increased acceptance of referrals  - Increased interaction with health and social services  - Survivors’ complex needs addressed  Final  - Reduced future IPV  - Improved mental health |
| **Immediate needs addressed** | | |
| **Resources** | **Reasoning** | **Outcomes** |
| - Access to resources that address immediate material needs  - Childcare facilities and provisions for children  - Emotional and instrumental support from networks | - Willingness to engage in interventions  - Increased comfort disclosing violence  - Improved coping strategies | Intermediary  - Increased help-seeking  - Increased receptivity and retention in mental health treatments  Final  - Improved psychological outcomes  - Reductions in IPV |
| **Resources** | **Reasoning** | **Outcomes** |
| - Systematic, institutional, financial and cultural barriers addressed  - Improved access to resources  - Ability to navigate relevant systems  - Increased social support | - Increased sense of empowerment and self-esteem  - Increased safety planning | - Increased quality of life  - Reduced symptoms of depression  - Reduced IPV |
| **Improved inter-agency collaboration** | | |
| **Resources** | **Reasoning** | **Outcomes** |
| - Increased access to resources  - Holistic model of care  - Extended reach for services  - Integration of treatment and prevention  - Ongoing appropriate support after discharge from healthcare services | - Negative impacts of multiple IPV-related traumas reduced | Intermediary  - Improved screening and detection rates  - Increased effectiveness of IPV response  - Increased service use  Final  - Reduced symptoms of PTSD |
| **Resources** | **Reasoning** | **Outcomes** |
| - Survivor and community norms and beliefs considered in relation to legal frameworks  - Culturally-appropriate responses centred on the survivor and community using either restorative justice approaches or perpetrator arrests | - Increased survivor agency and self-esteem | Intermediary  - Increased help-seeking from perpetrators, couples and survivors  - Behaviour change in perpetrators  Final  - Reduced IPV |
| **Financial Empowerment** | | |
| **Resources** | **Reasoning** | **Outcomes** |
| -Financial literacy, knowledge and skills in financial management  -Financial stability and independence  -Increased options to address situation  -Ability to recognise signs of economic abuse  -Ability to develop plans for education or employment  -Ability to build assets | -Sense of control  -Enhanced perception of, and actual, safety  -Reduced need for difficult trade-offs  -Increased financial self-efficacy and self-sufficiency  -Increased confidence, independence and pride  -Hope for the future | -Increased empowerment  -Increased financial and human capital |
| Interventions that increase a woman’s economic empowerment or independence, particularly when not combined with social intervention components to address elements such as safety planning, can increase the woman’s risk of violence in the home. This may be due to her reduced dependence on the perpetrator who may subsequently feel the need to exert his power over her, or a desire to gain access to these increased financial resources, which can be a source of conflict. This is exacerbated in settings with rigid gender norms where the empowerment of women may be perceived as a threat to men’s power and authority within the family and undermines his role as primary income generator. Increased financial resources may also encourage childbearing earlier in the relationship leading to increased dependence on the perpetrator, or may lead to an increase in the survivor’s heavy drinking. | | |
| **Resources** | **Reasoning** | **Outcomes** |
| -Greater economic well being  -Increased shared responsibility within household  -Reduced perceived threat from women’s income generation  -Increased social capital | -Increased self-esteem | Intermediary  -Reduced sexual risk behaviours  - increased empowerment  Final  -Reduced rates of STIS  -Reduced substance abuse  -Reduced IPV  -Reduced symptoms of depression |
| In settings with greater gender equality where women’s increased economic resources are viewed positively by both partners and afford a level of respect within the relationship, increased financial resources could create economic stability within the household, reducing stress and triggers for violence such as depression and alcohol dependence, as well as improving communication and problem solving between partners. Access to financial resources and women’s economic empowerment could also decrease the financial gap with their partner, increasing their independence and confidence, and reducing their willingness to tolerate violence and changes in their relationship dynamics, which ultimately reduces IPV risk. | | |
| **Resources** | **Reasoning** | **Outcomes** |
| -Increased access to economic resources  -Financial stability in wider household  -Decreased financial gap with partner  -communication and shared decision making in couple  -Increased ability to solve problems in couple  -Ability to recognise warning signs of violence behaviour | -Reduced stress for woman and perpetrator  -Greater negotiating power  -Greater respect within household  -Increased financial self-esteem and self-efficacy  -Increased confidence  -Change in women’s attitudes and identity  -Reduced dependency on partner  -Reduced willingness to tolerate problems in the relationship | Intermediary  -Increased economic, social and psychological empowerment for women  -Reduced triggers for violence (depression and substance use)  -Leaving or avoiding abusive relationship  Final  -Reduced risk of IPV |
| **Enhanced Safety, Control and Support** | | |
| **Developing a therapeutic alliance** | | |
| **Resources** | **Reasoning** | **Outcomes** |
| - Opportunities to disclose prior trauma and be listened to  - Positive and therapeutic relationship between provider and survivor  - Individual safety concerns prioritised  - Trust and rapport built | - Catharsis  - Perceptions of confidentiality and privacy  - Reduced feelings of isolation  - Increased sense of safety and stability | Intermediary  -Increased survivor confidence in the process and receptivity to interventions  - Improved personal and relationship developmental processes  Final  - Positive mental and physical health effects |
| **Increased agency** | | |
| **Resources** | **Reasoning** | **Outcomes** |
| - Opportunities to exercise agency and make choices throughout intervention and recovery process  - Ability to talk through solutions and discover own goals  - Choices respected by providers | - Confidence in ability to make changes  - Increased perceived support and trust in others  - Increased self-efficacy and readiness to exercise agency  - Increased use of safety behaviours | Intermediary  - Increased empowerment  Final  - Improved mental health  - Improved quality of life |
| **Increased safety planning** | | |
| **Resources** | **Reasoning** | **Outcomes** |
| - Immediate safety concerns addressed  - Economic safety plans developed  - Increased ability to manage finances and plan for the future | - Motivation to take action  - Reduced stress  - Increased self-efficacy | Intermediary  - Increased empowerment  - Reduced risks of HIV transmission  Final  - Reduced risks of violence  - Improved mental health |
| **Improved boundary setting, negotiating and communicating** | | |
| **Resources** | **Reasoning** | **Outcomes** |
| - Skills to manage boundaries  - Negotiating and communication skills  - Mediation between survivor and perpetrator by a neutral party as part of restorative justice work. | - Increased perceived and actual safety and feelings of control  - Reduced fear and distress  - Feels heard  - Healing and forgiveness | Intermediary  - Increased empowerment  Final  - Reduced PTSD symptomology  - Decreased depression  - Reduced anxiety  - Reductions in violence |
| An important moderator in the woman’s safety is her level of dependency on her partner and the abuser’s influence on her through ongoing contact or a continuing relationship. Continued or escalating abuse, including threatening of children, can increase women’s mental health problems, affect their ability to cope and prevent them from accessing treatment or achieving recovery goals. Women who are under the influence of the abuser may be reluctant to disclose violence or may minimise its extent. For women who are able to access treatment, perpetrator’s knowledge of this may be used as a catalyst to escalate abuse, turning her help seeking against her and undermining her credibility and ability to care for her children. | | |
| **Increased Knowledge** | | |
| **Increased acknowledgment and / or acceptance of the situation** | | |
| **Resources** | **Reasoning** | **Outcomes** |
| - Examining the dimensions of the problem  - Cognitive processing of traumatic events, habituating traumatic memories, debriefing  - Skills to manage grief | - Acceptance and acknowledgement of the situation  - Motivation to take action and seek help  - Feelings of relief  - Acknowledge and address traumatic experiences  - Understanding and sense-making  - Remembering, mourning and forgiveness | Intermediary  - Increased willingness to disclose violence  - Increase in social support  Final  -Reductions in anxiety, depression, PTSD  -Reductions in violence |
| **Increased awareness and psychoeducation** | | |
| **Resources** | **Reasoning** | **Outcomes** |
| - Increased knowledge about the causes and consequences of IPV  - Knowledge and awareness about resources  - Psychoeducation about PTSD and stress management | - Identifies patterns of abuse  - Motivation and strengthened resolve to take action  - Increased willingness to discuss safety plans | Intermediary  - Increased receptivity to interventions  Final  - Reductions in depression and anxiety symptoms  - Long term improvements in PTSD symptoms |
| **Increased understanding of women's rights** | | |
| **Resources** | **Reasoning** | **Outcomes** |
| - Knowledge and understanding of women’s rights and human rights in men and women  - Increased awareness of the problem at a community level  - Communication skills | - Improvement in attitudes that normalise violence in men and women  - Shifts in community gender equitable norms & beliefs, decreased acceptability of IPV | Intermediary  - Improved responses and support structures for survivors at a community level  - Increased social support  Final  - Reduced violence |
| Women’s ability to recognise and understand violent behaviours and abuse as problematic, rather than as acceptable or justifiable, can be influenced by social and cultural beliefs. This can lead to a lack of trust in, internal conflict over, and confusion from educational and awareness raising activities that do not align with these views and beliefs. | | |
| **Alterations to affective states and cognitions** |  |  |
| **Mood and emotional regulation** |  |  |
| **Resources** | **Reasoning** | **Outcomes** |
| - Stress management strategies and coping skills  - Stimulation of the parasympathetic nervous system  - Balance in the autonomic nervous system, improved perception, cognition & emotion regulation | - Ability to manage emotions and feelings  - Less reactive to stress and reduced stress triggers | Intermediary  - Increased safety behaviours  Final  - Reduced symptoms of PTSD, anxiety, depression |
| **Addressing guilt** | | |
| **Resources** | **Reasoning** | **Outcomes** |
| - Survivor guilt addressed  - Increased emotional safety and sense of control | - Reduced feelings of shame and self-blame  - Increased self-esteem and self-efficacy | Intermediary  - Increased receptivity and retention to interventions  Final  - Reduced symptoms of PTSD, depression  - Improved overall wellbeing |
| **Increased self-care and hope** | | |
| **Resources** | **Reasoning** | **Outcomes** |
| - Reconnection between self and body  - Normalisation of body talk  - Improvement of self-care activities | - Feels hope  - Improved emotional and physical intimacy  - Increased self-efficacy | Intermediary  - Empowerment  Final  - Improvements in depression severity, PTSD symptomology  - Improved quality of life |
| **Reframing inaccurate cognitions and changes in self-concept** | | |
| **Resources** | **Reasoning** | **Outcomes** |
| - Cognitive restructuring  - Thought-stopping skills  - Assessing and re-framing negative beliefs and inaccurate cognitions about the self | - Positive shifts in self-concept | - Reduced symptoms of depression, PTSD  - Reductions in psychological IPV |
| Interventions focused on shifting cognitions and affective states to improve the mental health of survivors are unlikely to be effective if they do not also ensure that women are supported in gaining access to the physical and social resources they need to mitigate the effects of traumatic stress caused by resource loss, which undermines survives coping, increases dependency on abusers, exacerbates PTSD symptoms, and increases psychological distress | | |
| **Increased skills to improve self-management** | | |
| **Increased problem solving / solution-seeking and goal setting** | | |
| **Resources** | **Reasoning** | **Outcomes** |
| - Problem solving and solution seeking skills  - Coping skills  - Decision-making skills  - Increased social support  - Ability to set and meet personal goals | - Increased self-esteem, self-efficacy  - Increased motivation to confront issues  - Increased physical and emotional safety and sense of control | Intermediary  - Empowerment  Final  - Reduced symptoms of anxiety, depression, PTSD  - Reduced IPV  - Increased life functioning  - Improved quality of life |
| When providers are not able to provide clear or effective solutions for survivors, either through challenges in screening, referral and service provision or through the process of goal setting and solution seeking itself, survivors may interpret this as an inability to intervene effectively. This could impact on survivor’s continued participation in interventions and possibly undermine solution-seeking components in interventions which aim to build self-efficacy and empowerment. | | |
| **Increased assertiveness and self-advocacy** | | |
| **Resources** | **Reasoning** | **Outcomes** |
| - Assertiveness and self-advocacy skills  - Communication skills | - Increased self-efficacy, self-esteem  - Empowered to take action  - Increased safety | Intermediary  - Reduced IPV risk  - Reduced HIV risk  Final  - Increased life functioning  - Reduced symptoms of PTSD and depression  - Increased emotional wellbeing and reduced distress |
| **Ability to manage symptoms, bodily sensations and triggers** | | |
| **Resources** | **Reasoning** | **Outcomes** |
| - Skills to manage body-mind relations  - Breathing regulation  - Stimulating vagal activity  - Centering and grounding  - Improved self-management skills  - Increased connection to body and ability to notice and identify bodily sensations  - Increased cognitive and behavioural skills to manage PTSD triggers | - Increased feelings of safety  - Sense of control | Intermediary  - Physiological relaxation  - Improved sleep quality  - Increased ability to manage trauma and stress, reduced stress reactivity  - Reduced trauma triggers  Final  - Reduced depression and anxiety  - Reduced PTSD symptoms  - Reduced IPV |
| **Improved Family and Social Relations** |  |  |
| **Improved connection and support structures** |  |  |
| **Resources** | **Reasoning** | **Outcomes** |
| - Recognise importance of social connections  - Increased trust and connection within networks  - Increased social support  - Increased access to resources, information, advice and experiential knowledge  - Ability to evaluate the situation and seek help  - Access to long term monitoring and follow-up | - Increased feelings of connection and perceived social support  - Feels protected, comfortable and understood  - Reduced shame and stigma  - Increased ability to cope  - Increased empowerment, self-worth, self-esteem  - Increased self-awareness  - Increased use of safety behaviours | Intermediary  - Increased participant engagement, uptake and retention of interventions  - Gains of more formal interventions maintained  Final  - Reduced symptoms of PTSD, depression, anxiety  - Improved quality of life  - Healing and thriving  - Reductions in future violence |
| This is particularly important for women with reduced social networks, such as marginalised or older women. For older women experiencing IPV, having a supportive family environment improves their ability to cope, and encouragement from family members (including children) leads to improved help seeking and participation in interventions. | | |
| **Improved mother-child relationships** | | |
| **Resources** | **Reasoning** | **Outcomes** |
| - Allow mothers to convey empathy for, and acceptance of, their children  - Allow mothers to understand and reflect on their child’s perspective  - Improved parenting skills  - Positive, reciprocal relationships between mothers and children  -Opportunities to share experiences, express upsetting feelings and jointly plan how to proceed | - Reduced child care concerns  - Confidence in their ability to parent  - Reduced feelings of isolation  - Reduced life stress  -Strengthened mother-child bond | Intermediary  - Improvements in child’s internalising symptoms  - Reduced family conflict  - Improved social support  Final  - Diminished distress  - Fewer symptoms of depression |
| In some circumstances, perpetrators may find ways to directly or indirectly undermine and disrupt interventions. Their deliberate use of children in abuse perpetration patterns can interfere with the mother-child relationship, affecting the woman’s self-worth and parenting confidence and altering her child’s perception of her [58], which could undermine the process of mother-child interventions. | | |
| Resource constraints on the side of the provider or the survivor could reduce the possibility of accessing or continuing IPV interventions, particularly those focused on improving family and social relations. For example, some mother-child interventions require a yearlong commitment to the programme which may be impossible when women are living in precarious situations, and shelters that provide refuge for families affected by violence may not have the means to provide structured and resource intensive programmes on a continual basis | | |
| **Altering relationship dynamics** | | |
| For survivors and perpetrators who do not wish to separate, interventions that encourage positive relationship behaviours and provide women with the skills to re-evaluate their relationships, allowing them to change the dynamics of psychological abuse by increasing their self-efficacy, readiness to take action, and safety behaviours, can lead to reductions in the occurrence of IPV. | | |
| **Resources** | **Reasoning** | **Outcomes** |
| - Cognitive restructuring  - Thought-stopping  - Problem-solving and coping skills  - Improved partner communication and strategies to reduce conflict | - Self-efficacy  - Readiness to take action  - Improved safety behaviours | - Reduce psychological IPV occurrence  - Reduced risk of IPV |
| Education and training interventions to improve partner support and relationship quality can change dysfunctional relationship patterns and reduce conflict through building communication skills and positive relationship behaviours. This can be protective against psychological and minor physical abuse. | | |
| **Resources** | **Reasoning** | **Outcomes** |
| - Healthy communication  - Identification and changes to dysfunctional relationship patterns and attitudes  - Improved ability to manage conflicts and aggression | - Improved relationship quality and satisfaction  - Improved inter-partner support  - Increased sexual safety behaviours | Intermediary  - Reductions in conflict  Final  - Protective against psychological and minor physical abuse |
